# The first comprehensive database of germline pathogenic variants in East Asian cancer patients

**DOI:** 10.1093/database/baab075

**Published:** 2021-12-29

**Authors:** Xiaoshun Shi, Ruidong Li, Jianxue Zhai, Allen Menglin Chen, Kailing Huang, Zhouxia Zheng, Zhuona Chen, Xiaoyin Dong, Xiguang Liu, Di Lu, Siyang Feng, Dingwei Diao, Pengfei Ren, Zhaoguo Liu, Grant Morahan, Kaican Cai

**Affiliations:** Department of Thoracic Surgery, Nanfang Hospital, Southern Medical University, 1838 Guang Zhou Avenue North, Guangzhou 510515, P. R. China; Harry Perkins Institute of Medical Research, QEII Medical Centre and Centre for Medical Research, The University of Western Australia, 6 Verdun St, Nedlands WA 6009, Australia; Genetics, Genomics, and Bioinformatics Program, University of California, 900 University Ave, Riverside, CA 92507, USA; Department of Thoracic Surgery, Nanfang Hospital, Southern Medical University, 1838 Guang Zhou Avenue North, Guangzhou 510515, P. R. China; Guangzhou Mendel Genomics and Medical Technology Co., Ltd., 6/F, Bldg D, 188 Kaiyuan Ave, Guangzhou 510535, P. R. China; Guangzhou Mendel Genomics and Medical Technology Co., Ltd., 6/F, Bldg D, 188 Kaiyuan Ave, Guangzhou 510535, P. R. China; Guangzhou Mendel Genomics and Medical Technology Co., Ltd., 6/F, Bldg D, 188 Kaiyuan Ave, Guangzhou 510535, P. R. China; Guangzhou Mendel Genomics and Medical Technology Co., Ltd., 6/F, Bldg D, 188 Kaiyuan Ave, Guangzhou 510535, P. R. China; Department of Thoracic Surgery, Nanfang Hospital, Southern Medical University, 1838 Guang Zhou Avenue North, Guangzhou 510515, P. R. China; Department of Thoracic Surgery, Nanfang Hospital, Southern Medical University, 1838 Guang Zhou Avenue North, Guangzhou 510515, P. R. China; Department of Thoracic Surgery, Nanfang Hospital, Southern Medical University, 1838 Guang Zhou Avenue North, Guangzhou 510515, P. R. China; Department of Thoracic Surgery, Nanfang Hospital, Southern Medical University, 1838 Guang Zhou Avenue North, Guangzhou 510515, P. R. China; Department of Thoracic Surgery, Nanfang Hospital, Southern Medical University, 1838 Guang Zhou Avenue North, Guangzhou 510515, P. R. China; Department of Thoracic Surgery, Nanfang Hospital, Southern Medical University, 1838 Guang Zhou Avenue North, Guangzhou 510515, P. R. China; Department of Thoracic Surgery, Nanfang Hospital, Southern Medical University, 1838 Guang Zhou Avenue North, Guangzhou 510515, P. R. China; Harry Perkins Institute of Medical Research, QEII Medical Centre and Centre for Medical Research, The University of Western Australia, 6 Verdun St, Nedlands WA 6009, Australia; Department of Thoracic Surgery, Nanfang Hospital, Southern Medical University, 1838 Guang Zhou Avenue North, Guangzhou 510515, P. R. China

## Abstract

Pathogenic germline variants in cancer-associated genes are risk factors for cancer predisposition. However, systematic mining and summarizing of cancer pathogenic or likely pathogenic variants has not been performed for people of East Asian descent. This study aimed to investigate publicly available data to identify germline variants in East Asian cancer cohorts and compare them to variants in Caucasian cancer cohorts. Based on the data we retrieved, we built a comprehensive database, named COGVIC (Catalog of Germline Variants in Cancer). A total of 233 variants in the East Asian population were identified. The majority (87%) of genes with cancer-associated variants were not shared between the East Asian and Caucasian cohorts. This included pathogenic variants in *BRCA2*. Our study summarized the prevalence of germline variants in East Asian cancer cohorts and provides an easy-to-use online tool to explore germline mutations related to cancer susceptibility.

**Database URL:**

http://www.cogvic.vip/

## Introduction

With the increasing application of next-generation sequencing (NGS) in basic research (1, 2) and clinical studies (3, 4), it is evident that NGS is a useful approach to investigate germline variants that predispose people to cancer. Although the number of germline variants associated with cancer susceptibility has expanded (5) and a considerable amount of exon sequencing data from tumor and tumor-matched control tissues are publicly available (6), systematic mining and summarizing of these pathogenic or likely pathogenic variants has not yet been performed.

Tumor susceptibility may differ between ethnic groups due to genetic differences (7). The Cancer Genome Atlas (TCGA) cooperative group provides large-scale cancer NGS data and predisposition variants in cancer (1), but the majority of its samples are from Caucasian subjects. Therefore, using the current TCGA data as a reference for cancer genetic comparisons in the East Asian population may be insufficient at best and even misleading due to underlying genetic differences between these populations.

To address this issue, we performed a comprehensive investigation of germline mutations in current genomic datasets, aiming to provide references for cancer genetic consulting for patients with ancestry from the East Asian geographical regions. We developed a novel germline mutation identifier for NGS data, established an online tool for germline mutation retrieval, described the prevalence of germline mutations in the East Asian population, and compared the prevalence of *BRCA2* hotspot variants in a larger worldwide population.

## Materials and methods

### Data collection

A comprehensive collection of NGS data released between 2012 and 2019 was obtained from NCBI SRA (Sequence Read Archive, https://www.ncbi.nlm.nih.gov/sra/), EBI (European Nucleotide Archive, https://www.ebi.ac.uk/ena) and DDBJ SRA (DNA Data Bank of Japan, https://www.ddbj.nig.ac.jp/dra/index-e.html). We also screened data from published sources, TCGA, and the International Cancer Genomics Consortium program. Only Illumina NGS datasets where raw data were available, including whole-genome sequencing (WGS), whole-exome sequencing (WES), and RNA sequencing (RNA-Seq) libraries, were included. Only studies that were approved by the original institutional review board were included. All data used were checked by scientists with a background in biology and confirmed by licensed doctors to ensure that they were derived from tumor studies. Data generated from normal samples, including peripheral blood and tumor-matched tissue, were first identified and then retrieved for subsequent in-house germline variant analysis. Based on data availability, the East Asian population in this study was defined as the eastern subregion of Asia, including China, Japan, Korea, and Vietnam.

### Development of a pipeline for variant identification

In this study, ‘variants’ included both single-nucleotide polymorphisms and insertion or deletion sequences (indels). A variant-identifying workflow named the ‘COGVIC ((Catalog Of Germline Variants In Cancer) analysis pipeline’ was developed for a comprehensive analysis to obtain a list of East Asian cancer-caused germline mutations.

#### Data quality control and preprocessing

FastQC software (8) was used for quality control of the raw sequencing data with default parameters. Adapter sequences and low-quality nucleotides were trimmed and filtered using Trim Galore software (version) (9) with the following parameters: -q 25—phred33—length 36—stringency 3—paired. The quality control step was run again on the cleaned data.

#### DNA data alignment and variant identification

For the genomics data generated with WGS-based, WES-based, and target-based technologies, alignment to the hg19/GRCH37 genome was performed with Bowtie2 software with the default settings (10). After stringent quality assessment and data filtering, reads with mapping quality greater than 20 were selected as high-quality reads for further analysis. Variants were detected with Varscan (—output-vcf 1—variants) based on a *P*-value of 0.01 (11).

#### RNA data alignment and variant identification

FastQC software was used for low-quality RNA-seq data filtering (8). STAR software (12) was used for RNA-seq data alignment to the GRCH38/hg38 reference genome. GATK was used for variant identification and detected with GATK (version 3.6.0).

#### Variant quality control

For each of the alignment results, an indexed BAM file was generated using SAMtools (13). Each BAM file was provided as input to each variant caller to generate a VCF file of unfiltered variant calls. To remove low-quality variants, genotypes were required to have DP >6, and all variants with quality scores of less than 40 were removed. To obtain further filtered variant calls, GATK was run through variant quality score recalibration steps (VQSR), as documented at the Broad Institute website (https://www.broadinstitute.org/gatk/guide/article?id=2805). Variants that passed both quality filters, i.e. those flagged as PASS in the GATK VQSR Filter, were used for the downstream analysis, including annotation and clinical interpretation.

#### Variant filtration and annotation

The annotation step was conducted with ANNOVAR (14) command for each subject (table_annovar.pl annovar/humandb -buildver hg19—remove—protocol refGene, avsnp150,1000g2015aug_all,1000g2015aug_eas, gnomad_exome_20190125,clinvar_20181225 -operation g, f, f, f,f,f). As common variants are usually easy to detect, we used ANNOVAR to filter the variants by minor allele frequency (MAF). All filtered and annotation criteria are listed below:

- Variants with MAF > 1% (GnomAD project, v2.1, 2018 release, East Asian population) were discarded.
- Synonymous, nonsplicing or nonexonic variants were discarded.
- Damaging missense mutations were defined as deleterious by at least two of the following criteria based on several function prediction models: SIFT (Sorting Intolerant From Tolerant) score ≤0.05, Polyphen2(HDIV) score ≥0.95, Mutation Assessor ≥2, Phred transformed CADD (Combined Annotation-Dependent Depletion) score ≥15, placental mammal PhyloP ≥ 2.4,and vertebrate PhyloP ≥4.
- Since we incorporated variants from clinical databases, such as ClinVar, COSMIC, TCGA, and OMIM, those variants associated with a phenotype (such as a disease or risk factor for a cancer-related disease) were kept for our final mutation list.

### Functional gene set analysis

We explored the key functions of the gene set mapped with pathogenic or likely pathogenic variants using g:Profiler (https://biit.cs.ut.ee/gprofiler/gost). The top 10 GO and KEGG pathways were plotted using the ggplot2 package in R.

### Online database

The online database was developed based on the widely used MediaWiki package. System requirements of MediaWiki are PHP 7.4.9+ and either MySQL 5.5.8+, MariaDB, SQLite or PostgreSQL.

## Results

### Study design

**Figure 1.**
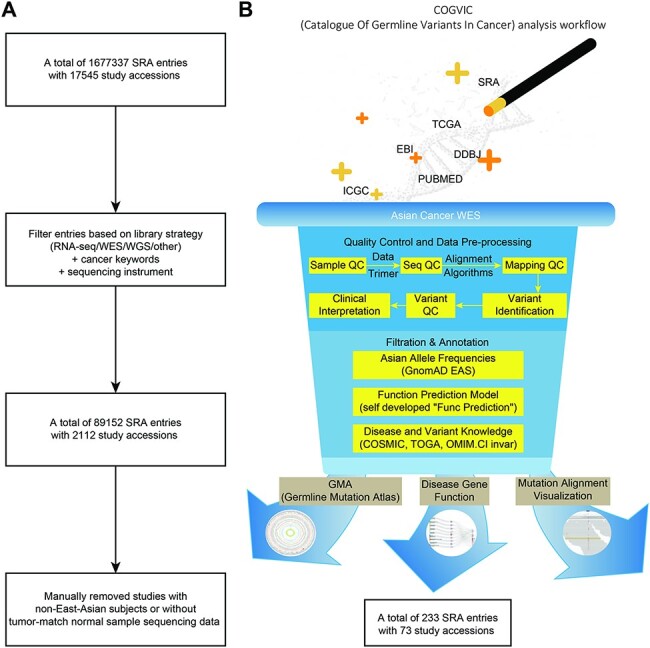
The COGVIC workflow of data selection and germline variant identification. (A) A total of 1 677 337 SRA entries with 17 545 study accessions were filtered out based on the inclusion criteria. (B) The pipeline of the COGVIC germline variant identifier.

We accessed the SRA database, excluding irrelevant items (e.g. noncancer study library construction strategies not related to RNA sequencing, WES or whole genome sequencing and non-Illumina HiSeq sequencing instruments) (Figure 1A). In addition, data from non-East Asian populations and data without tumor-matched controls were removed by manually reviewing the abstract or full text of each publicly available article or other data documentation. This process yielded 73 studies with WGS and WES data (Figure 1B). The datasets available for analysis were mainly from Singapore, Korea, and China (the COGVIC database, download page). A total of 1 677 337 entries and 2112 eligible studies with 89 152 sequencing items were collected.

### Clinical characteristics of patients in the included studies

Across these 73 studies, the average onset age was 54, ranging from 10 months to 85 years (in comparison, the TCGA cohort (5) established a diagnosis between 10 and 90 years of age). The COGVIC analysis workflow revealed a total of 233 potential cancer-causing germline variants (COGVIC variants) from 2401 tumor-matched normal samples across 20 types of cancer and 2 precancerous lesions. The cancer types and lesions with mutation frequencies in each group are listed in Table 1.

**Table 1.** The frequency of cancer pathogenic variants in the East Asian population

Cancer	Mutation frequency	Mutation cases/number of samples
Esophageal squamous cell carcinoma	9.1%	42/464
Gastric carcinoma	11.1%	26/234
Nasopharyngeal carcinoma	12.6%	26/206
Breast carcinoma	16.8%	32/190
Colorectal carcinoma	12.3%	19/154
Hepatocellular carcinoma	14.9%	22/148
Bladder cancer	3.8%	5/131
Cholangiocarcinoma	10.3%	13/126
Clear cell renal cell carcinoma	3.7%	4/108
Cervical cancer	9.8%	10/102
Pancreatic cancer	5.0%	5/101
Breast fibroepithelial tumors	4.4%	3/68
Lung cancer	9.4%	5/53
Oral squamous cell carcinoma	14%	7/50
Lymphoma	8%	2/25
Prostate cancer	10%	2/20
Follicular thyroid carcinoma	5.6%	1/18
Hepatoblastoma	16.7%	1/6
Acute myeloid leukemia	25%	2/5
Ovarian serous carcinoma	25%	1/4
Cervical intraepithelial neoplasia	2.0%	1/51
Esophageal precancerous lesion	8.3%	1/12
Other	2.4%	3/125

### Identification of pathogenic germline variants

The discovered germline variants were systematically compared against the dbSNP database (15), the Genome Aggregation Database (gnomAD), and the ClinVar database (16). All of the identified variants had been reported in dbSNP. We next analyzed the cancer contributing risk of these 233 germline variants discovered in different populations using gnomAD, which is a collection of germ cell mutations that can be used as a reference with data from different ethnicities, including ALL (world), AFR (African), AMR (Admixed American), ASJ (Ashkenazi Jewish), EAS (East Asian), FIN (Finnish), NFE (non-Finnish European), OTH (other) and SAS (South Asian). Except for variant data not given in gnomAD, odds ratio analysis of all the discovered variants reached a significant level as candidates for cancer susceptibility variants.

For clinical annotation of the COGVIC variants, we resorted to the ClinVar database, a standard, credible, and stable genetic variation-clinical phenotype association database. When inquiring about their clinical significance, we found that 82 out of 233 (35%) identified variants were not reported in the ClinVar database and that only 15 out of 233 (6.4%) variants were exactly matched with cancer predisposition. Furthermore, we found that some of the pathogenic variants we identified were associated in Caucasian subjects with benign diseases, such as deafness and benign recurrent intrahepatic cholestasis.

A total of 233 COGVIC variants were mapped to 89 genes. A list of the potential pathogenic variants and their characteristics can be accessed on the download page of the COGVIC database. Forty (17%) of these variants were associated with different cancer types in the East Asian population. For example, the variant rs62625308 (17) was reported to be associated with esophageal squamous cancer in a Chinese cohort but was associated with breast or ovarian cancer in the ClinVar database. Based on a further literature review for each COGVIC variant, rs28934578 was identified as a pathogenic variant of malignant esophageal tumors in both our dataset and the ClinVar database and reported as a variant significantly associated with the risk of breast cancer in an Indian cohort (18). These findings suggest that the COGVIC variants are potential pathogenic or likely pathogenic variants in the East Asian population. We also found an overall rate of 9.7% (233 cancer-associated variants in 2401 samples) pathogenic variants in the East Asian cohort and approximately 4.1% pathogenic variants and 3.8% likely pathogenic variants in the TCGA cohort (1).

### Differences in potential pathogenic genes between ethnicities

**Figure 2.**
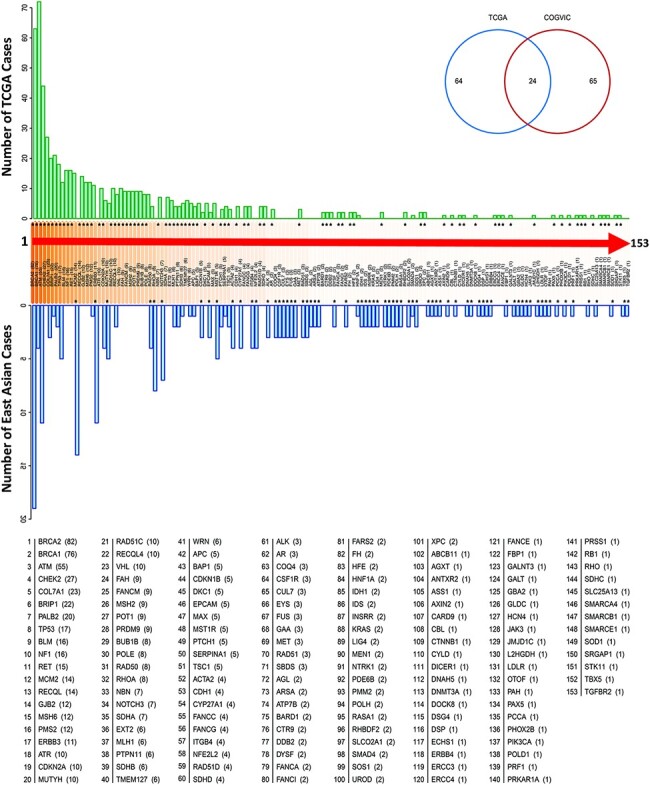
Genes with pathogenic variants identified in the COGVIC and TCGA cohorts. Distribution of the variants among 153 genes in both cohorts. The number of cases with pathogenic variants by gene in the TCGA database (green upper bars) is compared with those in the East Asian population (blue lower bars). Differences between the databases are indicated by asterisks above the gene name (when the number of TCGA cases is greater) or below the gene name (when the number of cases for the gene in the East Asian population is greater). The numbers in brackets beside the gene name are consistent with this system, i.e. asterisks above the number in the bracket indicate the number of TCGA cases, while asterisks below it indicate the number of cases in the East Asian population. The Venn diagram shows 64 genes with mutations unique to TCGA, 65 genes with mutations unique to the East Asian population and 24 genes with mutations detected in the two populations.

Our analysis showed that the enriched genes harboring pathogenic or likely pathogenic variants differed between the COGVIC East Asian population and TCGA Caucasian cohorts. Regarding pathogenic potency, we identified germline mutations that might strongly increase the risk of cancer susceptibility in East Asian populations, such as variants in Table 2. Genes with germline variants were not frequently reported as cancer predisposing genes in Caucasian cohorts. Furthermore, the top five most frequent genes with germline variants in East Asian subjects were *BRCA2* (8.2%), *MCM2* (6.0%), *ATM* (4.7%), *ERBB3* (4.7%) and *RHOA* (3.4%) (Figure 2). In comparison, the top five most frequent genes in the TCGA Caucasian cohort with pathogenic or likely pathogenic variants were *BRCA1* (*n* = 72/636, 11.3%), *BRCA2* (*n* = 63/636, 9.9%), *ATM* (*n* = 44/636, 6.9%), *CHEK2* (*n* = 27/636, 4.2%) and *BRIP1* (*n* = 21/636, 3.3%). Twenty-four genes overlapped between the two cohorts: *APC, ATM, BRCA1, BRCA2, BRIP1, CDKN1B, CDKN2A, COL7A1, EPCAM, HNF1A, MLH1, MUTYH, PALB2, PMS2, PTCH1, PTPN11, RAD50, RECQL4, SDHB, SERPINA1, SMAD4, TP53, TSC1* and *WRN*, suggesting that these genes contribute to cancer susceptibility worldwide (see the Venn diagram in Figure 2). We further observed that the genes with germline variants in the East Asian and TCGA Caucasian cohorts have different gene ontologies, reflecting the biological functions, pathways, or cellular localizations of these genes. In the COGVIC cohort, variants were enriched in genes functioning in the biological process of ontology and were involved in adenyl ribonucleotide binding, drug binding, and double-strand break repair (Figure S1a). Interestingly, the located genes with pathogenic or likely pathogenic variants in the TCGA Caucasian cohort were associated with DNA repair, cellular response to DNA damage stimulus, and DNA metabolic process pathways (Figure S1b). Additionally, KEGG pathway analysis revealed common cancer pathways in both the COGVIC cohort and the TCGA cohort, such as pathways in cancer, Fanconi anemia pathway, gastric cancer and colorectal cancer (Figure S1c and d). These findings indicate that further studies of the impacts of cancer-predisposing genes in Asian populations are needed.

**Table 2.** Germline variants that might strongly increase the risk of cancer susceptibility in East Asian populations

Gene	East_Asian_OR	95% CI	World_OR	95% CI
MCM2	22.7	[2.7, 188.2]	309.6	[31.5, 3048.8]
ERBB3	11.3	[1.2, 108.8]	154.7	[11.3, 2111.6]
CUL7	11.3	[1.2, 108.8]	77.3	[15.1, 395.8]

### The spectrum of *BRCA2* variants in East Asian and Caucasian cohorts

**Figure 3.**
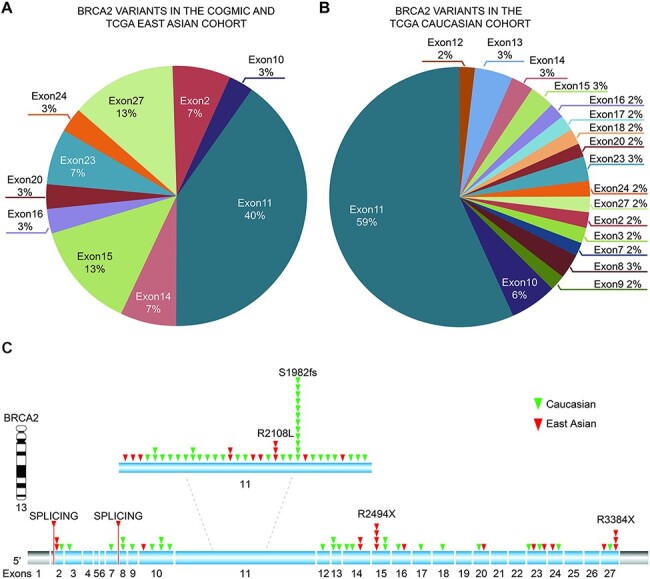
Distribution of identified pathogenic variants of BRCA2 around the world. (A) The proportion of BRCA2 variants in different exons in Asian cases from the COGVIC and TCGA cohorts. The colors represent different exons, e.g. exon 1 and exon 2. (B) The proportion of BRCA2 variants in different exons in the TCGA Caucasian cohort. The colors are consistent with the exons shown in A. (C) All variants placed on the BRCA2 gene map. The numbers indicate the exon numbers. The detected variants are indicated by arrows. Red arrows represent the East Asian population, whereas the green arrows represent the Caucasian population. There are two splicing mutations, one between exons 1 and 2 and one between exons 7–8.

We observed that the identified *BRCA2* variants were mainly located at exons 10 and 11 in all three cohorts, suggesting that this gene likely affects cancer susceptibility across the worldwide population (Figure 3A and B and Table 3). The top two exonic function changes in these variants were stop-gain and frameshift deletion. Of note, we did not find *BRCA2* variants in common between the East Asian (COGVIC and TCGA East Asian cohort) and TCGA Caucasian cohorts (Figure 3C), suggesting that each population has ethnic-specific variants in the *BRCA2* gene.

**Table 3.** The function and distribution of pathogenic BRCA2 variants in the COGVIC and TCGA cohorts

		COGVIC and TCGA Asian cohort	TCGA Caucasian cohort
Exonic functional change	Number of BRCA2 variants	23	63
	Synonymous SNV	2	
	Frameshift insertion	1	5
	Nonsynonymous SNV	10	2
	Unknown	3	3
	Stop gain	11	12
	Nonframeshift deletion	1	1
	Nonframeshift insertion		
	Frameshift deletion	1	40
	Frameshift substitution	3	
Pathogenicity classification	Benign		
	Benign/likely benign		
	Likely benign		
	Conflicting interpretations of pathogenicity	11	1
	Uncertain significance	6	
	Pathogenic/likely pathogenic	2	
	Pathogenic	9	61
	Unknown	4	1
			Number of variants
Variants in BRCA2 exon	Exon 1		
	Exon 2	2	1
	Exon 3		1
	Exon 4		
	Exon 5		
	Exon 6		
	Exon 7		1
	Exon 8		2
	Exon 9		1
	Exon 10	1	4
	Exon 11	12	37
	Exon 12		1
	Exon 13		3
	Exon 14	2	2
	Exon 15	4	2
	Exon 16	1	1
	Exon 17		1
	Exon 18		1
	Exon 19		
	Exon 20	1	1
	Exon 21		
	Exon 22		
	Exon 23	2	2
	Exon 24	1	1
	Exon 25		
	Exon 26		
	Exon 27	4	1
Variants in BRCA2 splicing site	Exon 1-2	1	
	Exon 7-8	1	

### Overview of the COGVIC database

**Figure 4.**
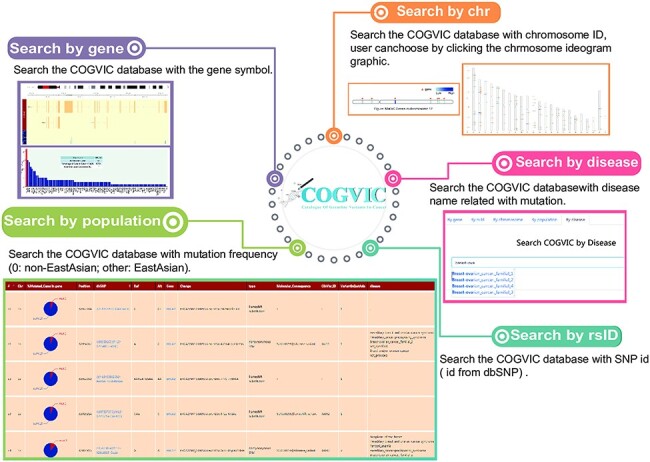
The main functions of the COGVIC database. This figure shows examples of outputs to specific search queries. Search by gene: results of search by gene symbol; Search by chr: search by chromosome ID; Users can choose by clicking the chromosome ideogram graphic. Search by rsID: uses SNP rs# from dbSNP; Search by population: retrieves population-specific information mutation frequency (0: not Asian; other: Asian); Search by disease: search with disease names finds associated mutations.

We built a user-friendly online tool for deposition, retrieval, and analysis of pathogenic and likely pathogenic germline mutations identified in the study (Figure 4). In the search section, users can query the database in five different ways: (i) Gene: searches by the gene symbol; (ii) Chromosome ID: searches using the chromosome ID, user can choose by clicking the chromosome ideogram graphic; (iii) SNP ID: searches with the rs# from dbSNP; (iv) Population: allows selection of population group (0: non-EastAsian; other: East Asian); (v) Disease: search by disease name related to mutation. The search terms are not case sensitive. Next, in the ‘browse’ section, users can consider all the information of germline mutations and can see the specific information of the mutation by clicking on the mutation of interest. The identified cancer pathogenic and likely pathogenic germline mutations can be retrieved from the download section, and newly submitted cancer pathogenic germline mutations will be updated after confirmation by a COGVIC administrator. Developers can request access to COGVIC data with API. An access license and access document can be obtained in this section.

## Discussion

The number of identified genes with germline mutations is a result of both pedigree analysis of target genes and populational gene analysis by NGS. Recent studies show that tumor susceptibility may differ between East Asians and Caucasians due to genetic differences (7). With increasing exome and WGS data published for the East Asian population, we have performed large-scale analyses of their germline variants. All these variants can be found using the online tools we developed and have made publicly available (http://www.cogvic.vip/). We identified 233 variants across 89 genes associated with cancer risk in this geographical region, with 24 genes being common to both East Asian and Caucasian populations. Importantly, our data showed that 9.7% of patients with cancer carry germline cancer susceptibility variants. This percentage was comparable to the 7.9% in the TCGA pancancer cohort. Because the majority of pathogenic variants in genes differed between the two populations, ethnic differences should be considered a factor in the investigation of cancer susceptibility genes.

Tumor onset may be related to ethnic differences due to genetic background (7). A previous study compared WES data from Asian (Chinese and Vietnamese) subjects with those from European Caucasians, finding seven genes with a high mutation rate based on a comparison with TCGA data, so these can be used for early screening of esophageal cancer risk in that geographical region (19). Among our identified genes with germline variants, only 27% overlapped with genes in the TCGA dataset. Biological function analysis indicated that these genes are associated with cancer predisposition in both cohorts. *BRCA2* harbored the most pathogenic or likely pathogenic variants in the East Asian cohort. Cancer pathogenic variants in *BRCA2* did not overlap significantly across cancer types between the COGVIC and TCGA cohorts, and it will be of interest to see if this will be confirmed in larger cohorts. Our findings suggest that the distribution of pathogenic or likely pathogenic germline mutations across cancers is different in the East Asian and TCGA Caucasian cohorts, providing evidence that the reference gene panel for genetic consultation should consider ethnicity as a factor.

Although some *BRCA2* variants were shared between Asian and Caucasian populations, the pathogenic role of a SNP can be ethnically specific, namely a carcinogenic SNP in European populations may have benign or even protective effects in Asian populations (20). However, the challenge of understanding the difference arises from the lack of data in these populations and previous findings from genome-wide association studies. Currently, our in-depth investigation of *BRCA2* variants revealed that *BRCA2* variants were not shared between ethnicities. Based on multiple cancer analyses, we postulated that the differences in the frequency and pathogenic role of variants between populations may not be limited to breast cancer. We further confirmed that common exons of *BRCA2*-harboring pathogenic variants are exons 10 and 11, as a previous study summarized (21). This may result from an increased probability of mutation due to the larger genomic size of these exons compared with the other exons in *BRCA2*. Therefore, SNP array-based cancer screening should include the SNPs we identified and consider ethnicity an important factor to avoid false-negative results.

There are some limitations of this study. First, although the study included a large-scale analysis, the open-access exon or WGS databases did not include data from all countries in the East Asian region either because these data have not been generated or are not publicly accessible. Thus, population bias may exist in our study. The second limitation is that, due to access issues, germline variants in the TCGA cohort cannot be downloaded; thus, the two datasets can only be indirectly compared. These problems confirm the importance of public access to germline variants under the context of ethical approval and personal confidentiality. Finally, the identified germline variants should be validated experimentally and supported by more clinical data in the future. We will update germline variants in the online COGVIC database annually as new data are published.

## Conclusions

Taken together, our results provide additional germline variants for genetic testing in the assessment of cancer risk, especially for East Asian countries and countries with people of East Asian descent. The results also support the notion of ethnicity-specific cancer susceptibility genes and provide targets that warrant further research for cancer prevention in different regions.

## Supplementary Material

baab075_Supp

## Data Availability

All the data are available online and documented at http://www.cogvic.vip/.
