# Supplementary figures and images for: The first comprehensive database of germline pathogenic variants in East Asian cancer patients
Source: Database (Oxford). 2021 Dec 29;2021:baab075. doi: 10.1093/database/baab075 (PMC8730286; doi:10.1093/database/baab075)

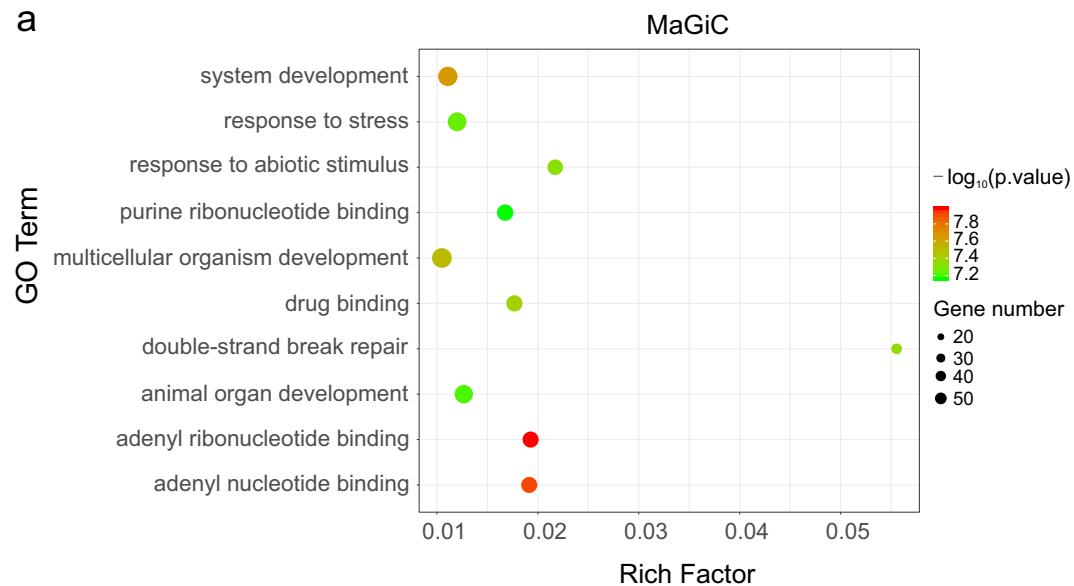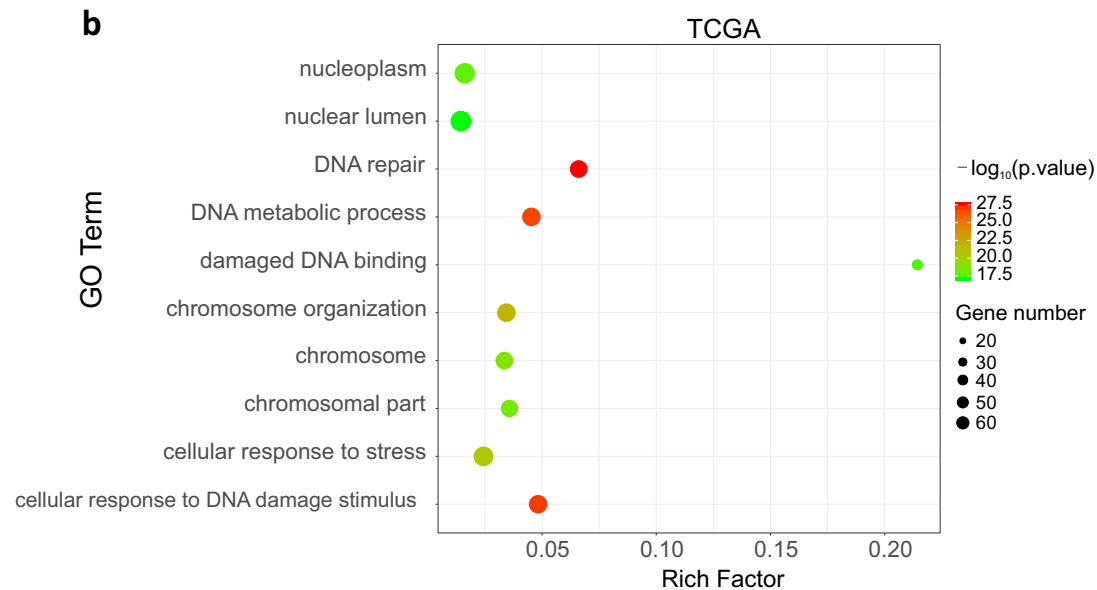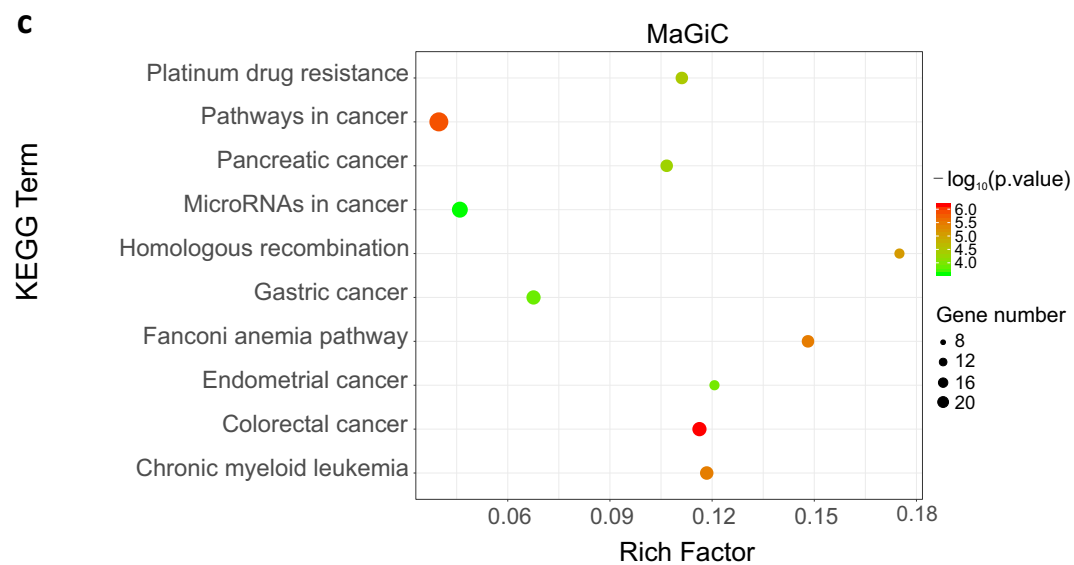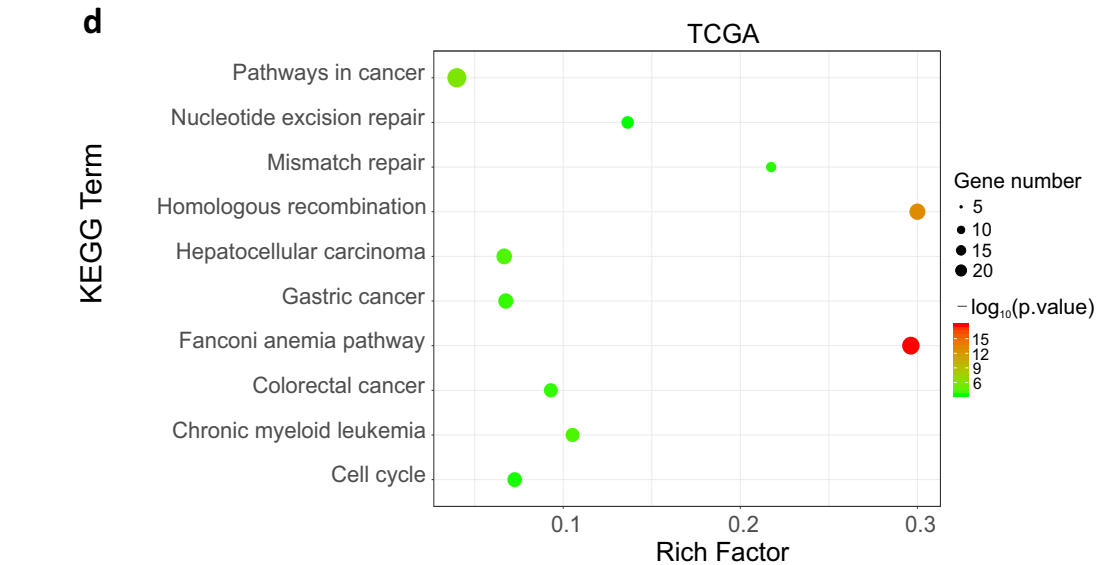

Supplement: baab075_Supp [file baab075_supp.zip › FigureS1 0918.pdf]
